# Correlation of Vitamin 25(OH)D, Liver Enzymes, Potassium, and Oxidative Stress Markers with Lipid Profile and Atheromatic Index: A Pilot Study

**DOI:** 10.3390/jox13020015

**Published:** 2023-04-01

**Authors:** Stavroula Ioannidou, Konstantina Kazeli, Hristos Ventouris, Dionysia Amanatidou, Argyrios Gkinoudis, Evgenia Lymperaki

**Affiliations:** 1 Department of Biomedical Sciences, International Hellenic University, 57400 Thessaloniki, Greece; 2 Department of Condensed Matter and Materials Physics, School of Physics, Faculty of Sciences, Aristotle University of Thessaloniki, 54124 Thessaloniki, Greece; 3 School of Veterinary Medicine, Aristotle University of Thessaloniki, 54124 Thessaloniki, Greece

**Keywords:** lipid profile, oxidative stress, liver enzymes, potassium, vitamin 25(OH)D

## Abstract

According to recent literature, there is a limited amount of data about the correlation of vitamin 25(OH)D, potassium (K), oxidative stress parameters, and other biomarkers with dyslipidemia, which is an established risk factor for cardiovascular diseases (CVDs). This study aims to investigate the correlation of lipid profile and atheromatic index TC/HDL with several biomarkers and oxidative stress parameters. A total of 102 volunteers, 67 with atheromatic index TC/HDL > 3.5 (Group A) and 35 with TC/HDL < 3.5 (Group B), aged from 26 to 78 years, participated in this study. Serum levels of triglycerides (TG), total cholesterol (TC), low- and high-density lipoproteins (LDL and HDL), vitamin 25(OH)D [25(OH)D], potassium (K), sodium (Na), lactose dehydrogenase (LDH), liver enzymes including serum glutamic oxaloacetic and glutamic pyruvic transaminases (SGOT and SGPT), gamma-glutamyl transferase (γ-GT), and alkaline phosphatase (ALP) were analyzed using standard photometric methods. Oxidative stress parameters such as reactive oxygen species (ROS) were detected with fluorometric methods, whereas total oxidative (TOS) and antioxidative status (TAS) were measured with spectrophotometric methods. According to the results, negative correlations of HDL (r = −0.593) and 25(OH)D (r = −0.340) and K (r = −0.220) were found, and positive expected correlations of LDL (r = 0.731), TC (r = 0.663), and TG (r = 0.584) with atheromatic index in the total studied sample were found. In conclusion, patients with a dyslipidemic profile should frequently check not only their lipid profile but also other biomarkers such as 25(OH)D, potassium, and oxidative stress markers to predict dyslipidemia and avoid subsequent disorders.

## 1. Introduction

Dyslipidemia is a well-known risk factor for cardiovascular diseases (CVDs) [1,2], which constitutes the leading cause of deaths globally [3]. Dyslipidemia is defined as an imbalance in the levels of lipids in the blood and is distinguished as either primary, with genetic determination, or secondary, due to acquired conditions [2]. This term fully expresses the most general disturbance of the metabolic balance of lipids [1]. In order to predict and diagnose dyslipidemia, there is a standard serum-based lipid profile test that includes the determination of total cholesterol (TC), high-density lipoprotein (HDL), low-density lipoprotein (LDL), and triglyceride (TG) levels [4]. With increased attention to LDL particle heterogeneity and particularly atherogenic forms of LDL such as small-dense LDL particles, oxidized LDL (ox-LDL), and lipoprotein (a), LDL remains the primary treatment target for coronary heart disease prevention. The atherogenic index (TC/HDL), as well as two new biomarkers, lipoprotein (a) and small-dense LDL, plays a significant role in predicting the risk of atherosclerosis and cardiovascular diseases [5,6]. An important role in lipoprotein metabolism is played by gonadal hormones, which may be related to cardiovascular risk affecting the plasma lipoprotein profile. Specifically, steroids with estrogenic activity increase the plasma levels of HDL and reduce the levels of LDL, whereas steroids with androgenic activity have the opposite effect. In addition, triglyceride levels are reduced by the exogenous administration of androgens and are increased by the oral intake of estrogens, even though this conflicts with the observed sex differences in this lipid [7,8]. Thus, the hormone-sensitive mechanisms are more critical for the metabolism of triglycerides, whereas the intracellular, cholesterol-sensitive mechanisms are more implicated in the metabolic regulation of cholesterol-rich lipoproteins and their derivatives [9].

Cholesterol synthesis in human cells occurs through the metabolic pathway involving 7-dehydrocholesterol. Moreover, vitamin D is either synthesized from 7-dehydrocholesterol with the contribution of UV-B radiation or obtained through food [10]. There are inactive forms that are converted into active forms through a biochemical pathway with the contribution of parathormone, which means that vitamin D is hormone dependent [11,12]. The fact that vitamin D deficiency is associated with an increased cardiovascular risk and cardiovascular risk factors forced us to investigate the correlation between 25-hydroxy vitamin D [25(OH)D] and lipid profiles in patients with dyslipidemic lipid profile [13]. Recent studies indicate a negative correlation between 25(OH)D and lipid profiles (especially TC, LDL, and TG) [14,15,16]. Moreover, a 2021 study confirmed the negative correlation between 25(OH)D levels and lipid profiles in non-obese children, especially in TG levels due to its involvement in lipolytic activity [15]. 

Additional factors such as liver enzymes, potassium ions, and oxidative stress appear to be associated with the lipid profile as well. Specifically, the serum levels of gamma-glutamyl transferase (γ-GT) show an independent correlation with serum lipids in dyslipidemic patients [17,18,19,20]. Thus, routine screening of liver enzymes and lipid profiles is recommended for the early detection of liver abnormalities, avoiding subsequent disorders of dyslipidemia. In addition, numerous studies have investigated the relationship between potassium (K) and lipid profiles. It seems that potassium is inversely associated with dyslipidemia risk factors [21,22]. Moreover, it was found that a high-cholesterol diet can affect potassium channels by decreasing their activity [23]. Dyslipidemia, mainly hypercholesterolemia, is associated with high-fat dietary consumption, which leads to high cholesterol in the vessels, triggers the formation of free radicals, and reduces antioxidant capacity [24]. Furthermore, even at an early age, there is a direct linear correlation between oxidative stress and serum lipid levels [25,26,27]. More specifically, a significant correlation between oxidized low-density lipoprotein and glutathione peroxidase (GPx) was found in a study, which helps to prevent lipid peroxidation and also maintains intracellular homeostasis as well as redox balance [27]. Therefore, there is a relationship between lipid profiles and redox status.

This study aims to investigate the correlation of 25(OH)D, K, liver enzymes, and oxidative stress parameters with serum lipids levels and the atheromatic index. These correlations have significant benefits as potential prognostic markers in early stages of dyslipidemia and could be useful for the management of the possible consequences of dyslipidemia.

## 2. Materials and Methods

### 2.1. Study Population

In this study, a total of 102 healthy volunteers’ samples were selected (51 males and 51 females, aged 26–78 years), who were examined at the General Hospital of Thessaloniki. The samples were divided into two groups, 67 (35 males and 32 females) with an atheromatic index TC/HDL > 3.5 (Group A) and 35 participants (16 males and 19 females) with an atheromatic index TC/HDL < 3.5 (Group B). All samples were collected between 8.00–9.00 a.m. and were centrifuged to collect serum samples as well as plasma samples from EDTA-treated blood. The serum and plasma samples were stored at −80 °C for 1 month. Hemolyzed samples were excluded. All of the participants were healthy, and they were not currently on any medication, supplement intake, or other drugs. Moreover, data were collected from all participants about sociodemographic characteristics, such as age and sex.

### 2.2. Analysis of Serum Lipoproteins, Vitamin D, Liver Enzymes and Electrolytes

All biochemical parameters, TC, HDL, LDL, TG, 25(OH)D, lactate dehydrogenase (LDH), liver enzymes serum glutamic oxaloacetic transaminase (SGOT), serum glutamic pyruvic transaminase (SGPT), γ-GT, alkaline phosphatase (ALP), and electrolytes [K, sodium (Na)] were measured with standard colorimetric assays by using an automated analyzer Abbott-Alinity ic-series. Specifically, the measurement of serum total cholesterol was detected using enzymatic colorimetric method (CHOD/POD). A hematogenous colorimetric assay was used for direct measurement of the HDL and LDL serum levels. The quantitation of TG serum levels was performed using an enzymatic colorimetric method (GPO/POD). The determination of 25(OH)D was calculated using a chemiluminescent microparticle immunoassay (CMIA). For the measurements of LDL, SGOT, SGPT, γ-GT, and ALP, kinetic colorimetric methods by IFCC were used. Moreover, the electrolytes (K and Na) were calculated with an Abbott-Alinity series analyzer with integrated chip technology (ICT) module based on a potentiometric method (ISE- Ion selective electrodes). All the samples were immediately analyzed and measured at least two times.

### 2.3. Analysis of Oxidative Stress Parameters (ROS, TOS, TAS) 

The levels of reactive oxygen species (ROS) were determined with a fluorometric method using a TECAN-infinite200 PRO fluorometer species and performed using the cell-permeable, ROS-sensitive probe 2′,7′-dichlorodihydrofluorescein diacetate (H_2_DCFDA) fluorescing at 520 nm (λ = 480 nm) upon oxidation. The fluorescence intensity is proportional to the ROS levels within the cell cytosol. All measurements of the ROS serum levels were estimated in accordance with the linear standard curve of hydrogen peroxide (H_2_O_2_) in a concentration range of 0–3 mM. The measurement of the total oxidative status (TOS) is based on the reaction of horseradish peroxidase (HRP) with serum oxidants resulting in the conversion of the tetramethylbenzidine (TMB) substrate into a colored blue product and is measured at 450 nm in a spectrophotometer. The quantification is performed with the delivered calibrator, in accordance with the linear standard curve of H_2_O_2_ in a concentration range of 0–0.5 mM. The determination of the total antioxidative status (TAS) is based on the reaction of exogenous H_2_O_2_ with serum antioxidants, which consumed part of the H_2_O_2_. The remaining H_2_O_2_ is determined photometrically by an enzymatic reaction, which converts TMB into a colored product, and is measured at 450 nm in a spectrophotometer. The difference of the sample values “with enzyme” and “without enzyme” is inversely proportional to the antioxidative capacity. The quantification is performed with the enclosed calibrator. All samples were analyzed immediately and measured at least two times.

### 2.4. Statistical Analysis 

The statistical analysis for the calculation of mean, standard deviation (SD), and all correlations were performed using SPSS tool version 22.0. Furthermore, student t-test and Pearson’s chi-square test were used to investigate the relationships between the two groups for each of the measured parameters. In all of the statistical analyses, the level of significance (*p*-value) was set at 0.001 and 0.05.

### 2.5. Ethical Considerations

The study was conducted in accordance with the Good Clinical Practice guidelines and the Declaration of Helsinki and the European General Regulation 2016/679 and law Ν.2472/1997. Ethical approval to perform this study was obtained from the Administration and the Scientific Council of the General Hospital of Thessaloniki Greece 24118/362022. The confidentiality of the participants was strictly preserved, and personal privacy was fully respected.

## 3. Results

The studied samples were divided into two groups: Group A (67 individuals) was identified as dyslipidemic lipid profile cases with TC/HDL > 3.5 (TC/HDL mean value = 4.97) and Group B (35 individuals) was the control group with TC/HDL < 3.5 (TC/HDL mean value = 2.96). From the determination of the lipids and lipoproteins, and comparing between the mean values of Group A and Group B, it was found that the mean values of the TC levels are 228 mg/dL vs. 163 mg/dL, HDL 48 mg/dL vs. 56 mg/dL, LDL 150 mg/dL vs. 87 mg/dL, and TG levels 164 mg/dL vs. 105 mg/dL, respectively. All of the above are statistically significant results with *p* < 0.0001. Moreover, the TC/HDL ratio shows statistically significant differences between the two groups with *p* < 0.0001, with Group A showing almost twice the TC/HDL value compared with Group B. In addition, Group A presents slightly lower mean values of 25(OH)D and Na, and a statistically significant decrease in K serum levels (*p* < 0.05). In Group A, all measured enzymes have an increased mean value, compared with Group B. Notably, the SGPT shows the most significant difference (*p* < 0.05). From the levels of oxidative stress indicators, a statistically significant decrease in TAS can be observed (*p* < 0.05) alongside a slightly increase in ROS and TOS, and therefore there is also an increase in the TOS/TAS index. In more detail, the mean values of ROS levels are 1.34 mM in Group A versus 1.27 mM in Group B, TOS levels are 170 μmol/L in Group A versus 165 μmol/L in Group B, TAS levels are 224 μmol/L in Group A versus 244 μmol/L in Group B and TOS/TAS levels are 0.8 in Group A versus 0.7 in Group B. The mean values and standard deviation of the parameters measured in this study are summarized in Table 1.

The following Table 2, Table 3, Table 4, Table 5 and Table 6 present the correlation between all biomarkers and lipid profiles in the two groups as well as in the total samples.

### 3.1. Correlation between Atheromatic Index (TC/HDL) and Lipid Profile

We observed a moderate negative correlation between the TC/HDL ratio and the HDL (r = −0.593) in the total cases. However, there is a strong positive correlation of. the LDL (r = 0.731), and a moderate positive correlation of TC (r = 0.662) and TG (r = 0.583) with the atheromatic index (Table 2).

### 3.2. Correlation between Vitamin D and Lipid Profile

The vitamin D serum level has a weak negative linear relationship with TC (r = −0.089) and LDL (r = −0.056). Moreover, it is significantly negatively related to the TG level (r =−0.455) and TC/HDL ratio (r = −0.340) in the total subjects. In addition, there is a positive statistically significant correlation with HDL levels (r = 0.327) in the total group (Table 3).

### 3.3. Correlation of Liver Enzymes and LDH with Lipid Profile

Serum-liver enzyme correlation is found without statistical significance in the total group. Nevertheless, we observe a significant negative correlation of LDH with TC (r = −0.190) and TG (r = −0.321) in the total group, with TC (r = −0.276), LDL (r = −0.212), TG (r = −0.333), and with TC/HDL (r = −0.249) in Group A. In Group B, we find a statistically significant negative correlation of LDH only with TG (r = −0.467). Moreover, of all liver enzymes, only SGPT is significantly positive correlated with lipid profile. In particular, SGPT is positively associated weak with TC (r = 0.135), LDL (r = 0.117), and moderate TG (r = 0.311). No statistically significant differences are observed in other liver enzymes with the lipid profile (Table 4).

### 3.4. Correlation between Electrolytes and Lipid Profile

Serum K levels are significantly inversely associated with TC/HDL (r = −0.220) but there is significantly positively correlation to the lipid profile, notably with HDL (r= 0.254) in the total group. Respectively, there is a significant negative correlation of K and TC/HDL (r = −0.298) and a positive correlation of K and HDL (r = 0.307) in Group A. On the other hand, serum Na levels correlation are found without statistical significance in the total group. There is a significant negative correlation of Na with TC (r = −0.343), LDL (r = −0.345), and TC/HDL (r = −0.173) in Group B, but it there is a significantly positive correlation with TC (r = 0.270), LDL (r = 0.214), and TC/HDL (r = 0.115) in Group A (Table 5).

### 3.5. Correlation between Oxidative Stress Parameters and Lipid Profile

There is a significantly negative correlation only of TAS with TC (r = −0.375), HDL (r = −0.233), and LDL (r = −0.403) in the total group. In the Group B, there is a significantly positive correlation of TOS and TOS/TAS with LDL (r = 0.224 and r = 0.335, respectively), but there is a significantly negative correlation between TOS and LDL (r = −0.356). We observe a negative strong correlation between TOS/TAS levels and TC/HDL (r = −0.352) ratio in Group B. However, there is an inverse correlation between ROS (r = −0.283) and TC in dyslipidemic lipid profile cases (Table 6).

## 4. Discussion

Dyslipidemia is a major contributor to cardiovascular morbidity and mortality. Although awareness of the importance of the risk of dyslipidemia has increased, the prevention and prognosis of dyslipidemia have not improved accordingly. This study aims to investigate the correlation between 25-hydroxy vitamin D [25(OH)D], liver enzymes, potassium (K), and oxidative stress markers in dyslipidemic lipid profile. These parameters could be used combined with lipid profile as a prognostic marker in the early stages of dyslipidemia to avoid its subsequent disorders. In addition, we confirm the already-known correlation of atheromatic index with the lipid profile and its important role as a potential prognostic marker for dyslipidemia and cardiovascular diseases (CVDs) [28,29]. As expected, there is a significantly negative correlation between TC/HDL ratio levels and serum high-density lipoprotein levels (r = −0.593), whereas a statistically significant positive correlation of low-density lipoprotein (r = 0.731), total cholesterol (r = 0.662), and triglycerides (r = 0.583) with atheromatic index can be seen, in total cases, in our research. It is worth noting that atheromatic index TC/HDL is considered to be a significant cumulative marker of the presence of atherogenic dyslipidemia [30].

New studies indicate that vitamin D plays a particular role in developing atherosclerosis [10]. Lipid metabolism is partially regulated by vitamin D because hepatocyte receptors for vitamin D are integral in the regulation of cholesterol transport. Moreover, vitamin D has an anti-inflammatory role, reducing insulin resistance by decreasing low-grade chronic inflammation, lowering triglycerides, and increasing serum high-density lipoprotein levels [14,31]. In vitamin-D-deficient patients, cholesterol transport becomes altered, leading to an increase in circulating cholesterol levels, which can lead to atherosclerosis development [31]. In clinical trials, supplementation in deficient patients has been found to reduce the levels of total cholesterol, triglycerides, and low-density lipoprotein in serum, but not the levels of high-density lipoprotein correlation [10,16]. Our study is in accordance with these reports regarding the correlation of 25-hydroxy vitamin D serum levels with the lipid profile. In particular, our results reveal a negative correlation of the TC/HDL ratio with total cholesterol, low-density lipoprotein, and triglycerides serum levels, whereas there is a positive correlation of the TC/HDL ratio with high-density lipoprotein levels. These results are aligned with Glueck et al.’s research that serum 25-hydroxy vitamin D possibly acts as a protective factor against cardiovascular diseases due to the inverse correlation of serum 25-hydroxy vitamin D with low-density lipoprotein and triglycerides, and also due to the significant positive relationship between 25-hydroxy vitamin D and high-density lipoprotein in patients with dyslipidemia [14].

It is a reasonable assumption that liver disfunction can affect lipid levels due to the fact that the liver plays an important role in lipid metabolism [32]. Specifically, fatty acids accrue in the liver by hepatocellular uptake from the plasma and by de novo biosynthesis. Fatty acids are either metabolized by oxidation within the cell or secreted into the plasma within triglyceride-rich, very low-density lipoproteins [33,34]. Therefore, an abnormal lipid profile can be reasonably expected in those with severe liver dysfunction. In our research, we did not observe any significant correlation between the studied groups in liver enzymes and lipid profile. This result may occur because we did not include patients with liver disease or liver abnormalities. However, Dahman et al. recommended the use of glutamic pyruvic transaminase (SGPT) and gamma-glutamyl transferase (γ-GT) serum levels as a potential prognostic marker for non-alcoholic fatty liver disease in Type 2 diabetes mellitus patients with dyslipidemia [19], precisely because there was a liver dysfunction in their study group. Moreover, Kathak et al. found a strong positive correlation between dyslipidemia and liver enzymes, especially in correlation of gamma-glutamyl transferase levels [20]. Concerning our results, liver enzymes do not appear to have a strong statistical correlation with lipid profile, but there are decreased gamma-glutamyl transferase serum levels in patients with TC/HDL > 3.5 and increased levels of the other enzymes, including serum glutamic oxaloacetic transaminase (SGOT), serum glutamic pyruvic transaminase, alkaline phosphatase (ALP), and lactate dehydrogenase (LDH). Another serum enzyme, lactate dehydrogenase, which is an enzyme with wide tissue distribution in the body, especially in the liver, heart, kidney, skeletal muscle, and erythrocytes, plays an important role in the glycolytic path [35]. In our study, serum lactate dehydrogenase levels show a significant negative correlation, especially with triglycerides serum levels and are found to be elevated in the group with an increased atheromatic index. This finding is in agreement with another study that observed hypocholesterolemia and elevated triglycerides levels caused by muscle lipolysis in calves whose lactate dehydrogenase fractions revealed a significant increase in LDH4 and LDH5 activity and a decrease in LDH1 activity when they were electrophoretically separated [35].

The correlation between serum electrolytes and the lipid profile is multifactorial, depending on several other factors, which include age and associated conditions [36]. Our results show that serum potassium (K) levels are inversely related to the TC/HDL index because there is a significant positive correlation with high-density lipoprotein serum levels in the total group. This result may be due to an independent effect of serum potassium on lipolysis and fatty acid production according to Menni et al. [37]. In addition, this result could be useful to investigate the effect of potassium supplements on the lipid profile, also including the atheromatic index TC/HDL. Our finding about the inverse correlation of potassium and TC/HDL ratio is broadly in line with other studies, reporting the positive effect of increased potassium supplement intake resulting in reducing cardiovascular risk factors, such as hypertension and dyslipidemia [38,39,40]. On the other hand, serum sodium (Na) levels show no significant correlation with the lipid profile. One of the main reasons for our result is likely because sodium levels are strongly affected by dietary salt intake [41]. In fact, it is claimed that the role of salt in the digestion of lipid emulsions suggests that the addition of sodium chloride in emulsified fats leads to a reduction in electrostatic repulsion between lipid particles, thus increasing the formation of ‘bridged’ lipid clusters [42]. Our results agree with a previous study of Menni et al. that observed significant correlations only of serum potassium levels with individual fatty acid metabolites and specific enrichment of fatty acid pathways [37]. However, the effect of supplementation of potassium intake and the use of electrolytes generally in the lipid profile and also in dyslipidemia remain unknown, thus further research is necessary.

Interestingly, we observed that high values of reactive oxygen species (ROS), total oxidative status (TOS) and TOS/TAS ratio are found in patients with dyslipidemic lipid profile contrary to healthy participants. Turkdogan et al. confirmed a significant correlation between oxidative stress and serum lipid levels in healthy young people [25]. Furthermore, Viktorinova et al. found a statistically notable correlation between oxidative stress markers and lipid profile and suggested their use as early prevalence markers of atherosclerosis-related diseases, especially in the healthy population [26]. It is known that increased levels of reactive oxygen species lead to the oxidation of low-density lipoproteins and produce oxidized low-density lipoprotein (ox-LDL) particles. Specifically, oxidized low-density lipoprotein is absorbed in macrophages via scavenger receptor pathways to form cholesteryl ester-rich foam cells and making endothelial cells dysfunctional, in part by taking up oxidized low-density lipoprotein via the lectin-like oxidized low-density lipoprotein receptor [43]. Previous studies have shown that the role of oxidized low-density lipoprotein, especially in the oxidation of lipoproteins, results in an imbalance between pro- and antioxidant systems, involved in the pathologic process of atherosclerosis, by changing cellular functions [44,45]. Thus, further studies should focus on mechanism in order to block the production of reactive oxygen species, contributing to the prevention of the oxidation of low-density lipoproteins and atherosclerosis as well. According to the literature, our results reveal, as expected, a positive strong correlation of low-density lipoprotein with total oxidative status and TOS/TAS, and there is a significantly negative correlation between low-density lipoproteins and the total antioxidative status (TAS), in Group B [44,45]. However, there is a strong negative correlation of TOS/TAS ratio with the atheromatic index in healthy participants. Thus, TOS/TAS could be used as a prognostic marker in healthy people and in the early stages of dyslipidemia, although it would be necessary to conduct more clinical trials with a larger number of participants.

According to the literature, the prevalence of dyslipidemia increases with age. Moreover, several health behaviors can lead to a dyslipidemic lipid profile, specifically, a sedentary lifestyle with excessive dietary intake of calories, saturated fat, cholesterol, and trans fats. Taking the above into consideration, we selected samples of a healthy population. Furthermore, both of the study groups included participants who had the same age range, weight and race. Thus, we tried to limit unobserved covariates and other factors which possibly could influence the research.

Our research indicates that there might be a significant correlation of 25-hydroxy vitamin D, potassium, and reactive oxygen species with lipid profile in participants with a high atheromatic index. In order to investigate this possible correlation, future supplementary studies should be designed to reinforce this correlation and provide comprehensive insights.

## 5. Conclusions

Based on our results, we suggest that it is important to understand how lipid metabolism can affect a lack of certain biomolecules such as K and 25(OH)D to prevent side effects of dyslipidemia. Liver enzymes and TOS/TAS need to be further studied as laboratory indicators in the prevention of the subsequent disorders of dyslipidemia and atherosclerosis as well. Concerning the study of oxidative stress, we recommend the measurement of the TOS/TAS ratio, as it expresses the total oxidizing capacity and the total antioxidant status; thus it is a more reliable indicator, and it should be checked regularly combined with other biomarkers in prevention. According to this study, there is not a strong statistical correlation of liver enzymes with the lipid profile, but there are decreased γ-GT serum levels in patients with TC/HDL > 3.5 and increased levels of the other enzymes, including (SGOT, SGPT, ALP and LDH). Finally, further research, which could study a larger number of samples, could strongly support the observed correlation of K, vitamin D, liver enzymes, and oxidative stress markers in individuals with dyslipidemic lipid profile.

## Figures and Tables

**Table 1:** Mean and SD values of biomarkers according to their atheromatic index.

Markers	Reference Values	TC/HDL > 3.5 (n = 67) Group A	TC/HDL < 3.5 (n = 35) Group B	Total TC/HDL (n = 102)	*p*-Values
		Mean (±SD)	Mean (±SD)	Mean (±SD)	
TC	<200 mg/dL	228 (±36.7)	163 (±40.4)	205 (±48.9)	<0.0001
HDL	>45 mg/dL	48 (±10.3)	56 (±15.4)	51 (±12.9)	<0.0001
LDL	<130 mg/dL	150 (±36.4)	87 (±32.1)	128 (±46.2)	<0.0001
TG	<150 mg/dL	164 (±78.0)	105 (±43.8)	144 (±73.7)	<0.0001
TC/HDL	<3.5	4.9 (±1.3)	2.9 (±0.4)	4.3 (±1.4)	<0.0001
25(OH)D	>30 ng/mL	20 (±9.2)	22 (±9.0)	21 (±9.1)	<0.1
SGOT	5–45 U/L	21 (±10.3)	19 (±6.4)	20 (±9.2)	<0.1
SGPT	0–55 U/L	24 (±10.0)	20 (±7.6)	23 (±9.4)	<0.05
γ-GT	9–36 U/L	30 (±20.5)	32 (±31.5)	30 (±24.2)	<0.1
LDH	125–220 U/L	197 (±75.6)	184 (±53.6)	193 (±70.0)	<0.1
ALP	40–150 U/L	71 (±20.6)	67 (±13.9)	70 (±18.7)	<0.1
K	3.5–5 mmol/L	4.39 (±0.4)	4.43 (±0.5)	4.4 (±0.5)	<0.05
Na	136–148 mmol/L	141 (±3.2)	142 (±2.2)	141 (±2.9)	<0.1
ROS	1.25 mM	1.34 (±1.22)	1.27 (±0.73)	1.31 (±1.07)	<0.1
TOS	180–310 μmol/L	170 (±77)	165 (±79)	169 (±77)	<0.1
TAS	280–320 μmol/L	224 (±27)	244 (±50)	229 (±35)	<0.05
TOS/TAS	0.5–1	0.8 (±0.3)	0.7 (±0.4)	0.8 (±0.3)	<0.1

Abbreviations. TC: total cholesterol, HDL: high-density lipoprotein, LDL: low-density lipoprotein, TG: triglycerides, TC/HDL index, 25(OH)D: 25-hydroxy vitamin D, SGOT: serum glutamic oxaloacetic transaminase, SGPT: serum glutamic pyruvic transaminase, γ-GT: gamma-glutamyl transferase, LDH: lactate dehydrogenase, ALP: alkaline phosphatase, K: potassium, Na: sodium, ROS: reactive oxygen species, TOS: total oxidative status, TAS: total antioxidative status, TOS/TAS index.

**Table 2:** Pearson’s correlation coefficient (r) between atheromatic index (TC/HDL) and Lipid profile.

Marker	TC/HDL		
Lipid Profile	Group A (r)	Group B (r)	Total (r)
TC	0.514	0.169	0.663
HDL	−0.686	−0.493	−0.593
LDL	0.578	0.337	0.731
TG	0.486	0.456	0.584

Abbreviations TC: total cholesterol, HDL: high-density lipoprotein, LDL: low-density lipoprotein, TG: triglycerides, TC/HDL index.

**Table 3:** Pearson’s correlation coefficient (r) between Vitamin D and Lipid profile.

Marker	25(OH)D		
Lipid Profile	Group A (r)	Group B (r)	Total (r)
TC	−0.061	0.030	−0.089
HDL	0.489	0.175	0.327
LDL	0.006	0.064	−0.056
TG	−0.456	−0.444	−0.445
TC/HDL	−0.467	−0.197	−0.340

Abbreviations TC: total cholesterol, HDL: high-density lipoprotein, LDL: low-density lipoprotein, TG: triglycerides, TC/HDL index, 25(OH)D: 25-hydroxy vitamin D.

**Table 4:** Pearson’s correlation coefficient (r) of Liver enzymes and LDH with Lipid profile.

			MARKER				
			SGOT (r)	SGPT (r)	γ-GT (r)	ALP (r)	LDH (r)
Lipid Profile	Group A	TC	−0.173	0.053	0.009	0.042	−0.276
		HDL	0.199	0.058	−0.015	−0.146	0.073
		LDL	−0.165	0.021	−0.0005	0.126	−0.212
		TG	−0.076	0.271	0.041	−0.099	−0.333
		TC/HDL	−0.254	0.051	0.038	0.076	−0.249
	Group B	TC	0.111	−0.008	0.220	0.497	0.004
		HDL	0.228	−0.063	0.226	0.220	−0.107
		LDL	0.008	−0.053	0.069	0.487	0.159
		TG	0.063	0.259	0.363	0.187	−0.467
		TC/HDL	−0.123	0.164	0.052	0.435	0.198
	Total	TC	−0.036	0.135	0.063	0.111	−0.190
		HDL	0.158	−0.044	0.126	−0.074	0.005
		LDL	−0.050	0.117	−0.005	0.155	−0.114
		TG	−0.027	0.311	0.102	−0.065	−0.321
		TC/HDL	−0.117	0.170	0.004	0.141	−0.128

Abbreviations TC: total cholesterol, HDL: high-density lipoprotein, LDL: low-density lipoprotein, TG: triglycerides, TC/HDL index, SGOT: serum glutamic oxaloacetic transaminase, SGPT: serum glutamic pyruvic transaminase, γ-GT: gamma-glutamyl transferase, ALP: alkaline phosphatase, LDH: lactate dehydrogenase.

**Table 5:** Pearson’s correlation coefficient (r) between electrolytes and Lipid profile.

Markers	K (r)			Na (r)		
Lipid Profile	Group A	Group B	Total	Group A	Group B	Total
TC	0.172	0.126	0.023	0.270	−0.343	−0.020
HDL	0.307	0.155	0.254	0.141	−0.162	0.056
LDL	0.181	0.028	−0.017	0.214	−0.345	−0.013
TG	−0.137	0.221	−0.083	0.086	−0.163	−0.002
TC/HDL	−0.298	−0.044	−0.220	0.115	−0.173	−0.024

Abbreviations TC: total cholesterol, HDL: high-density lipoprotein, LDL: low-density lipoprotein, TG: triglycerides, TC/HDL index, K: potassium, Na: sodium.

**Table 6:** Pearson’s correlation coefficient (r) between oxidative stress parameters and Lipid profile.

			MARKER			
			ROS (r)	TOS (r)	TAS (r)	TOS/TAS (r)
Lipid Profile	Group A	TC	−0.283	−0.147	−0.214	−0.065
		HDL	−0.000002	−0.121	−0.257	−0.056
		LDL	−0.237	−0.064	−0.302	0.038
		TG	−0.114	−0.121	0.344	−0.203
		TC/HDL	−0.191	0.063	0.150	0.039
	Group B	TC	−0.185	0.111	−0.417	0.241
		HDL	−0.239	0.245	−0.311	0.351
		LDL	−0.116	0.224	−0.356	0.335
		TG	−0.090	−0.543	−0.319	−0.457
		TC/HDL	0.185	−0.377	−0.170	−0.352
	Total	TC	−0.174	−0.027	−0.375	0.078
		HDL	−0.075	−0.007	−0.233	0.067
		LDL	−0.130	0.0007	−0.403	0.117
		TG	−0.091	−0.120	0.045	−0.157
		TC/HDL	−0.094	0.043	−0.083	0.058

Abbreviations TC: total cholesterol, HDL: high-density lipoprotein, LDL: low-density lipoprotein, TG: tri-glycerides, TC/HDL index, ROS: reactive oxygen species, TOS: total oxidative status, TAS: total antioxidative status, TOS/TAS index.

## Data Availability

No data available due to privacy and ethical restrictions.

## Abbreviations

ALP	alkaline phosphatase
CVDs	cardiovascular diseases
γ-GT	gamma-glutamyl transferase
HDL	high-density lipoprotein
HRP	horseradish peroxidase
H_2_DCFDA	2′,7′-dichlorodihydrofluorescein diacetate
H_2_O_2_	hydrogen peroxide
GPx	glutathione peroxidase
K	potassium
LDH	lactate dehydrogenase
LDL	low-density lipoprotein
Na	sodium
Ox-LDL	oxidized low-density lipoprotein
ROS	reactive oxygen species
SD	standard deviation
SGOT	serum glutamic oxaloacetic transaminase
SGPT	serum glutamic pyruvic transaminase
TAS	total antioxidative status
TC	total cholesterol
TG	triglycerides
TMB	tetramethylbenzidine
TOS	total oxidative status
25(OH)D	25-hydroxy vitamin D
